# Tumor-Stroma Ratio and Programmed Cell Death Ligand 1 Expression in Preoperative Biopsy and Matched Laryngeal Carcinoma Surgical Specimen

**DOI:** 10.3390/ijms23148053

**Published:** 2022-07-21

**Authors:** Lara Alessandrini, Leonardo Franz, Marta Sbaraglia, Tommaso Saccardo, Filippo Cappello, Alessandro Drigo, Anna Chiara Frigo, Gino Marioni

**Affiliations:** 1 Department of Medicine DIMED, University of Padova, Via Giustiniani 2, 35128 Padova, Italy; lara.alessandrini@aopd.veneto.it (L.A.); marta.sbaraglia@aopd.veneto.it (M.S.); flppcappello@gmail.com (F.C.); 2 Department of Neuroscience DNS, Otolaryngology Section, University of Padova, Via Giustiniani 2, 35128 Padova, Italy; leonardo.franz@aopd.veneto.it (L.F.); tommaso.saccardo@aopd.veneto.it (T.S.); alessandro.drigo.1@studenti.unipd.it (A.D.); 3 Guided Therapeutics (GTx) Program International Scholar, University Health Network (UHN), Toronto, ON M5G2C4, Canada; 4 Department of Cardiac-Thoracic-Vascular Sciences and Public Health, University of Padova, Via Giustiniani 2, 35128 Padova, Italy; annachiara.frigo@unipd.it

**Keywords:** laryngeal squamous cell carcinoma, tumor-stroma ratio, programmed cell death ligand 1, PD-L1, biopsy, prognosis

## Abstract

Programmed cell death ligand 1 (PD-L1) seems to rely on close relations between neoplastic and immune cells in the tumor microenvironment. Tumor to stroma ratio (TSR) has been associated with prognosis in different malignancies. The aims of this exploratory investigation were to analyze for the first time the: (i) association between TSR, PD-L1 expression and other clinical–pathological features in laryngeal squamous cell carcinoma (LSCC) biopsies and paired surgical specimens; (ii) prognostic and predictive role of TSR and PD-L1. TSR, PD-L1 expression (in terms of combined positive score [CPS]), and other clinical–pathological features were analyzed in biopsies and surgical specimens of 43 consecutive LSCC cases. A CPS < 1 evaluated on surgical specimens was associated with a low TSR (stroma rich) on both biopsies and surgical specimens (*p* = 0.0143 and *p* = 0.0063). Low TSR showed a significant negative prognostic value when evaluated on both biopsies and surgical specimens (HR = 8.808, *p* = 0.0003 and HR = 11.207, *p* = 0.0002). CPS ≥ 1 appeared to be a favorable prognostic factor (HR = 0.100, *p* = 0.0265). The association between bioptic and surgical specimen TSR and PD-L1 expression should be further investigated for a potential impact on targeted treatments, also with regard to immunotherapeutic protocols.

## 1. Introduction

Over the past 30 years, survival rates for laryngeal squamous cell carcinoma (LSCC) patients have not improved, despite the introduction of new surgical approaches and the adoption of chemotherapy and radiation treatment strategies for organ preservation [1,2]. Therefore, pathological and oncological research on LSCC, in particular on advanced ones, has focused on novel biomarkers that could better stratify patients’ prognosis to allow appropriate and tailored treatments [3,4,5].

In the last decade, the tumor interaction with the stroma, the main microenvironment component, has been extensively investigated because of its fundamental role in different stages of tumorigenesis [6]. In order to quantify this component, recent studies have identified several measurable parameters on histological sections, including tumor to stroma ratio (TSR). TSR is an easy, cost-effective technique, applied on hematoxylin and eosin (H&E)-stained slides, estimating the proportion of tumor and its surrounding stroma. The TSR assessment method has recently been standardized and a cut-off value of 50% has been suggested to stratify tumors with low or high stromal content [7]. The latter has been associated with worse prognosis in different organs/sites malignancies [8]. TSR has shown to have a good inter-observer reproducibility [7,8] and is currently considered a promising predictor of prognosis also in head and neck malignancies [9], including laryngeal carcinoma [10,11,12,13]. Moreover, other parameters that are probably linked to tumor–stroma interaction, such as the type of stroma (fibrotic or desmoplastic), tumor growth pattern, neoplastic nest size and the presence of tumor budding, preliminarily showed a prognostic role in head and neck carcinoma [10,14,15].

Similarly, the cross-talk between cancer and immune cells in the tumor microenvironment has relevant prognostic and therapeutic implications. Programmed cell death ligand 1 (PD-L1) is a crucial molecule in the immune checkpoint biological pathway [16]. PD-L1 clinical value has also been proven to be both a prognostic marker and a target for therapy in a wide range of human malignancies, including head and neck carcinoma [17]. On the strength of the KEYNOTE-048 trial survival results, pembrolizumab monotherapy or pembrolizumab in combination with platinum +5-fluorouracil was approved in 2019 by the European Medicines Agency as first-line treatment for patients with recurrent or metastatic head and neck carcinoma whose tumors express PD-L1 with a combined positive score (CPS) ≥1 [18]. Just a few studies investigated the role of PD-L1 focusing only on patients with LSCC; the CPS method-already in use for assessing PD-L1 in other malignancies was very rarely adopted for LSCC [19]. PD-L1 biological relations with tumor-infiltrating lymphocytes (TILs) have recently been explored in LSCC [19].

The aims of the present exploratory investigation were to analyze: (I) the concordance between LSCC biopsies and paired surgical specimens in terms of characterization of TSR, stroma type, large cell nests and tumor budding; (II) the association between TSR, PD-L1 expression and other clinical–pathological features; (III) the prognostic and predictive role of TSR and PD-L1 in a consecutive series of LSCC.

## 2. Results

### 2.1. Overall Outcomes

Fourteen out of the 43 consecutive patients with LSCC considered in this series (32.6%) experienced disease recurrence after a mean follow-up of 11.9 months (median 11 months). Twenty-nine (67.4%) showed no evidence of disease after a mean of 84.7 months (median 84 months).

### 2.2. Concordance between Biopsies and Paired Surgical Specimens in Terms of TSR and Other Pathological Variables

**Figure 1:**
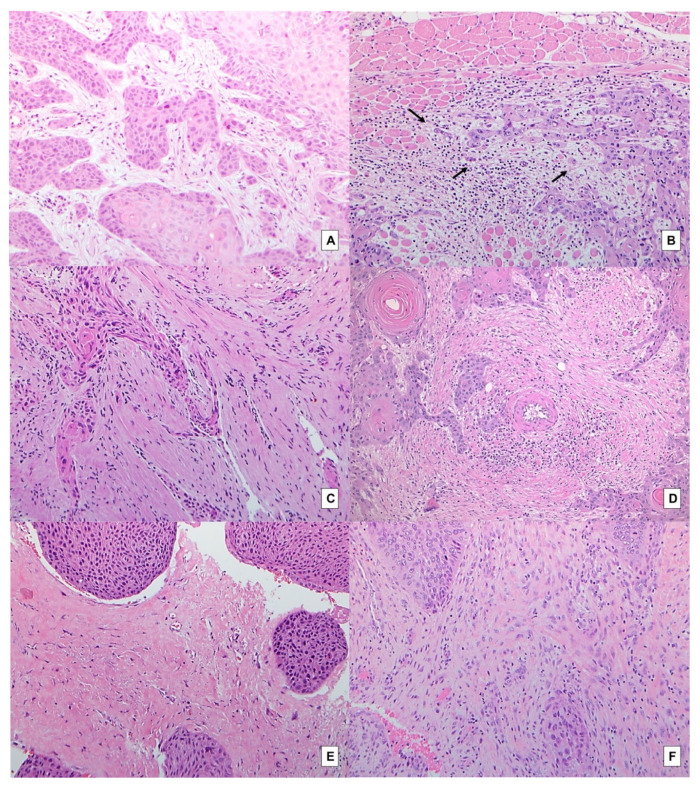
Paired biopsies and surgical specimens: (**A**) LSCC biopsy with a high TSR (stroma-poor case), displaying tumor buds in a fibroblastic stroma (H&E, original magnification 100×); (**B**) surgical specimen of the same case as A, in which tumor buds are evident at the front of invasion of the tumor (arrows) in a fibroblastic stroma; smooth muscle can be seen at the top of the image (H&E, original magnification 100×); (**C**) biopsy of a low TSR (stroma-rich case) (H&E, original magnification 100×); (**D**) paired surgical specimen (H&E, original magnification 100×); (**E**) biopsy of low TSR (stroma-rich case), large cell nests and fibrotic stroma (H&E, original magnification 100×); (**F**) paired surgical specimen (H&E, original magnification 100×).

Table 1 shows the concordance between biopsies and paired surgical specimens in terms of characterization of TSR, stroma type, large cell nests and tumor budding. According to Altman’s benchmark scale [20], a good agreement between biopsies and surgical specimens was found for both TSR (Figure 1C–F) and large cell nests (Figure 1E,F) (AC1 statistic 0.7957 and 0.6935, respectively), and a very good agreement for both stroma type (Figure 1A–F) and tumor budding (Figure 1A,B) (AC1 statistic 0.8829, and 0.8244, respectively).

### 2.3. Association between TSR and Other Clinical–Pathological Variables on Both Biopsies and Paired Surgical Specimens

Table 2 reports the analysis of the association between TSR and the considered clinical–pathological variables on biopsies and related surgical specimens.

**Figure 2:**
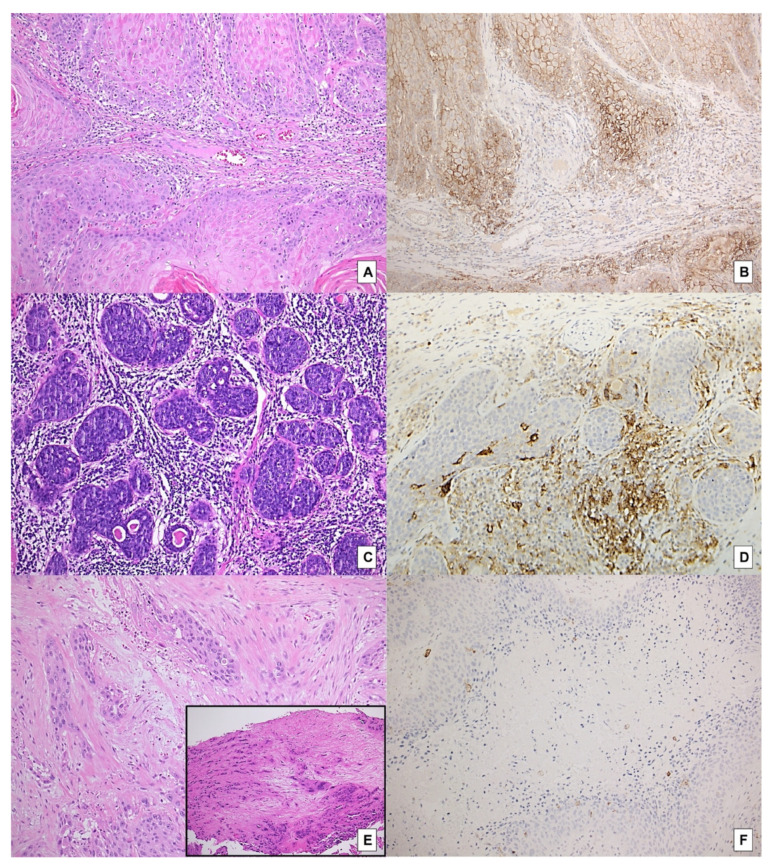
Association between TSR and other clinical–pathological variables on both biopsies and paired surgical specimens: (**A**) surgical specimen with a high TSR (stroma-poor case) (H&E, original magnification 100×); (**B**) same case, showing a high expression of PD-L1 (CPS > 1), mainly by neoplastic cells (immunostaining for PD-L1, original magnification 100×); (**C**) another TSR- high case associated with high density of stromal TILs (H&E, original magnification 100×); (**D**) same case, showing a high expression of PD-L1 (CPS > 1), mainly by mononucleated inflammatory peritumoral cell (immunostaining for PD-L1, original magnification 100×); (**E**) a stroma-rich case (TSR low) on both biopsy (inset) and associated surgical specimen (H&E, original magnification 100×); (**F**) A stroma rich case displaying a nearly absent PD-L1 expression (CPS < 1) (immunostaining for PD-L1, original magnification 100×).

A CPS < 1 evaluated on surgical specimens was found to be significantly associated with a low TSR (stroma rich) on both biopsies and surgical specimens (*p* = 0.0143, and *p* = 0.0063, respectively) (Figure 2A–F).

Statistical trends towards an association between TSR in surgical specimens and TILs (*p* = 0.0660) (Figure 2C,D), pattern of invasion (*p* = 0.0774) and tumor budding on surgical specimens (*p* = 0.0767) were disclosed. Furthermore, TSR and peri-tumoral budding count, both determined on surgical specimens, were mutually associated (*p* = 0.0464) (Figure 2E).

**Table 2:** Association between clinical–pathological variables and TSR on both biopsies and surgical specimens.

	TSR (Biopsy)		*p* Value *	TSR (Surgical Specimen)		*p* Value *
	TSR High/Stroma Poor (N = 30)	TSR Low/Stroma Rich (N = 13)		TSR High/Stroma Poor (N = 29)	TSR Low/Stroma Rich (N = 14)	
**Stroma type (biopsy)**						
Fibroblastic	3 (10.0%)	2 (15.4%)	0.6299			
Fibrotic	27 (90.0%)	11 (84.6%)				
**Large cell nests (biopsy)**						
Absent	6 (20.0%)	3 (23.1%)	1.0000			
Present	24 (80.0%)	10 (76.9%)				
**Budding count, intratumoral (biopsy)**						
Mean (SD)	1.47 (2.92)	0.85 (1.46)	0.8217			
Median (IQR)	0.00 (0.00–1.00)	0.00 (0.00–1.00)				
**Tumor budding (biopsy)**						
Low risk	27 (90.0%)	12 (92.3%)	1.0000			
High risk	3 (10.0%)	1 (7.7%)				
**Stroma type (surgical specimen)**						
Fibroblastic	3 (10.0%)	2 (15.4%)	0.6299	2 (6.9%)	3 (21.4%)	0.3091
Fibrotic	27 (90.0%)	11 (84.6%)		27 (93.1%)	11 (78.6%)	
**Large cell nests (surgical specimen)**						
Absent	5 (16.7%)	3 (23.1%)	0.6806	4 (13.8%)	4 (28.6%)	0.4038
Present	25 (83.3%)	10 (76.9%)		25 (86.2%)	10 (71.4%)	
**Budding count, peritumoral (surgical specimen)**						
Mean (SD)	1.53 (2.29)	3.46 (5.32)	0.3892	1.14 (1.64)	4.14 (5.29)	**0.0464**
Median (IQR)	0.50 (0.00–3.00)	1.00 (0.00–4.00)		0.00 (0.00–2.00)	3.00 (0.00–6.00)	
**Tumor budding (surgical specimen)**						
Low risk	27 (90.0%)	10 (76.9%)	0.3455	27 (93.1%)	10 (71.4%)	0.0767
High risk	3 (10.0%)	3 (23.1%)		2 (6.9%)	4 (28.6%)	
**CPS (surgical specimen)**						
< 1	15 (50.0%)	12 (92.3%)	0.0143	14 (48.3%)	13 (92.9%)	**0.0063**
≥1	15 (50.0%)	1 (7.7%)		15 (51.7%)	1 (7.1%)	
**TILs (surgical specimen)**						
Mean (SD)	35.67 (21.12)	28.08 (20.87)	0.2470	37.41 (21.45)	25.00 (18.29)	0.0660
Median (IQR)	30.00 (15.00–50.00)	20.00 (15.00–30.00)		35.00 (20.00–50.00)	20.00 (15.00–30.00)	
**Pattern of invasion (surgical specimen)**						
Expansile	23 (76.7%)	7 (53.8%)	0.1630	23 (79.3%)	7 (50.0%)	0.0774
Infiltrative	7 (23.3%)	6 (46.2%)		6 (20.7%)	7 (50.0%)	

* Fisher’s exact test for categorical variables; Mann-Whitney test for quantitative variables. TSR: tumor-to-stroma ratio; CPS: PD-L1 continuous positive score; TILs: tumor infiltrating lymphocytes; SD: standard deviation; IQR: inter-quartile range. Significant *p*-values are in bold.

### 2.4. Prognostic Value of TSR and Clinical–Pathological Variables

Table 3 summarizes the results of the univariate Cox regression model for DFS prediction, based on the variables considered in this study. The 8th edition of the TNM Classification of Malignant Tumors was considered [21].

Low TSR (stroma rich) also showed a significant negative prognostic value, when evaluated on both biopsies and surgical specimens (HR = 8.808 95% CI: 2.739–28.323, *p* = 0.0003, and HR = 11.207 95% CI: 3.093–40.611, *p* = 0.0002, respectively). On the other hand, a CPS ≥ 1 appeared to be a favorable prognostic factor (HR = 0.100 95% CI: 0.013–0.764, *p* = 0.0265).

**Table 3:** Prognostic value of clinical–pathological variables (univariate Cox’s regression model).

N = 43	Outcome		*p* Value	
	No Recurrence (N = 29)	Recurrence (N = 14)		HR (95% CI)
**Age**				
Mean (SD)	63.48 (8.69)	68.29 (6.29)		
Median (IQR)	64.00 (60.00–68.00)	68.00 (64.00–72.00)	0.0772	1.061 (0.994–1.134)
**pT classification**				
T1 + T2	15 (51.7%)	4 (28.6%)		1
T1	6 (20.7%)	*1 (7.1%)*		
T2	9 (31.0%)	*3 (21.4%)*		
T3 + T4	14 (48.3%)	10 (71.4%)	0.1869	2.185 (0.684–6.977)
T3	8 (27.6%)	*9 (64.3%)*		
T4	6 (20.7%)	*1 (7.1%)*		
**Grading**				
G1	5 (17.2%)	2 (14.3%)		1
G2 + G3	24 (82.8%)	12 (85.7%)	0.7986	1.215 (0.272–5.440)
G2	16 (55.2%)	*4 (28.6%)*		
G3	8 (27.6%)	*8 (57.1%)*		
**N-status**				
N0	24 (82.8%)	8 (57.1%)		1
N+	5 (17.2%)	6 (42.9%)	0.0736	2.642 (0.911–7.657)
**Stage**				
I + II	13 (44.8%)	4 (28.6%)		1
I	6 (20.7%)	*1 (7.1%)*		
II	7 (24.1%)	*3 (21.4%)*		
III + IV	16 (55.2%)	10 (71.4%)	0.3525	1.734 (0.543–5.533)
III	8 (27.6%)	*4 (28.6%)*		
IV	8 (27.6%)	*6 (42.9%)*		
**TSR (biopsy)**				
TSR high/Stroma poor	26 (89.7%)	4 (28.6%)		1
TSR low/Stroma rich	3 (10.3%)	10 (71.4%)	**0.0003**	8.808 (2.739–28.323)
**Stroma type (biopsy)**				
Fibroblastic	3 (10.3%)	2 (14.3%)		1
Fibrotic	26 (89.7%)	12 (85.7%)	0.7941	0.819 (0.183–3.662)
**Large cell nests (biopsy)**				
Absent	7 (24.1%)	2 (14.3%)		1
Present	22 (75.9%)	12 (85.7%)	0.3873	1.937 (0.433–8.671)
**Budding count, intratumoral (biopsy)**				
Mean (SD)	1.45 (2.97)	0.93 (1.44)		
Median (IQR)	0.00 (0.00–1.00)	0.00 (0.00–2.00)	0.4730	0.904 (0.687–1.190)
**Tumor budding (biopsy)**				
Low risk	26 (89.7%)	13 (92.9%)		1
High risk	3 (10.3%)	1 (7.1%)	0.6632	0.636 (0.083–4.868)
**TSR (surgical specimen)**				
TSR high/Stroma poor	26 (89.7%)	3 (21.4%)		1
TSR low/Stroma rich	3 (10.3%)	11 (78.6%)	**0.0002**	11.207 (3.093–40.611)
**Stroma type (surgical specimen)**				
Fibroblastic	4 (13.8%)	1 (7.1%)		1
Fibrotic	25 (86.2%)	13 (92.9%)	0.4897	2.049 (0.268–15.675)
**Large cell nests (surgical specimen)**				
Absent	5 (17.2%)	3 (21.4%)		1
Present	24 (82.8%)	11 (78.6%)	0.9029	0.924 (0.257–3.317)
**Budding count, peritumoral (surgical specimen)**				
Mean (SD)	1.41 (2.34)	3.57 (5.02)		
Median (IQR)	0.00 (0.00–2.00)	2.00 (0.00–4.00)	0.0992	1.095 (0.983–1.219)
**Tumor budding (surgical specimen)**				
Low risk	26 (89.7%)	11 (78.6%)		1
High risk	3 (10.3%)	3 (21.4%)	0.5232	1.516 (0.423–5.438)
**CPS (surgical specimen)**				
<1	14 (48.3%)	13 (92.9%)		1
≥1	15 (51.7%)	1 (7.1%)	**0.0265**	0.100 (0.013–0.764)
**TILs % (surgical specimen)**				
Mean (SD)	37.24 (21.28)	25.36 (18.96)		
Median (IQR)	30.00 (20.00–50.00)	20.00 (10.00–30.00)	0.0795	0.972 (0.942–1.003)
**Pattern of invasion (surgical specimen)**				
Expansile	23 (79.3%)	7 (50.0%)		1
Infiltrative	6 (20.7%)	7 (50.0%)	0.1074	2.368 (0.829–6.766)

Significant *p*-values are in bold.

## 3. Discussion

### 3.1. Significance of TSR as a Marker of Biological Aggressiveness in LSCCs

Only a few previous studies [10,11,12] investigated the role of TSR in patients with LSCC. Moreover, some studies [10,11] did not consider only LSCCs, but grouped together different tumor sites (pharynx) and included preoperative biopsies only in cases treated with neo-adjuvant chemotherapy, as a surrogate for surgical specimens [11]. In this study, TSR has been evaluated for the first time on both preoperative biopsies and paired surgical specimens of LSCC, showing a good concordance between the two sources of neoplastic tissue (AC1 statistic 0.796), and a high prognostic predictive value in terms of relapse. A similar comparison was performed previously only by a study on esophageal carcinoma, in which the TSR score on biopsies was concordant with surgical specimens in 81% of the cases [22].

Considering surgical specimens, an association between low TSR (stroma-rich tumors) and high tumor budding has been found. This is consistent with recent studies on colon cancer [23,24] and LSCC [10] and reflects the recently accepted view of both variables being markers of epithelial–mesenchymal transition (EMT). As is well-known, EMT is fundamental for carcinogenesis and shows prognostic implications also in LSCCs [25]. A mesenchymal (stromal) phenotype has been associated with an invasive front of the tumor where cancer cells (tumor budding) interface with stromal cells [26]. Accordingly, from a pathological viewpoint, a high tumor budding activity has been significantly associated with stroma-rich tumors in head and neck cancer [10,12], and it has been speculated that higher amounts of stroma could facilitate EMT. Considering the role of inflammatory infiltrate, stroma seems to promote tumorigenesis by preventing immune cell infiltration in the tumor. The stromal myofibroblasts/fibroblasts create a physical barrier against immune cells due to their contractile properties, hence promoting tumor progression [27]. This peculiar mechanism seems to emerge also from our study: the stroma-rich cases, evaluated in surgical specimens, had a lower percentage of stromal TILs (even though not statistically significant *p* = 0.0660), suggesting that the tumor microenvironment could determine an increased difficulty for immune cells to reach the tumor core. This particular behavior between TSR and TILs, as already proven in different tumor histotypes [28,29], appears to be one of the possible immune-escaping mechanisms that cancer cells establish during EMT to elude immunosurveillance. These findings are aligned with previous observations that high stroma percentage is associated with a weaker peri-tumoral inflammatory infiltrate in patients with other solid malignancies [28]. Interestingly, the Tumor Immune Dysfunction and Exclusion (TIDE) prediction score [30], a surrogate for increased immune evasion potential, was found to be higher in stroma-high cases versus the stroma-low ones [31].

From a biological point of view, TSR seems to be the histological epiphenomenon of the genomic concept of the Stromal-Epithelial Signature Ratio (SESR), which depends on the differential expression of patterns of genes specific for fibroblasts (C3, CFP, ECM1, THBS1 and TIMP1), and epithelial malignant cells (C4BPA, CFB, CHGA, PF4, PPBP, SAA2, SERPINA1 and SERPIND1), respectively [31]. In line with this view, the enrichment scores for the stromal signature were found to be higher in the stroma-high tumors, compared to the stroma-low tumors, probably reflecting the EMT biological process [32].

### 3.2. PD-L1 and TSR

The relationship between the tumor and immune microenvironment is reflected also by the expression of PD-L1, which is a crucial molecule in the regulation of the immune response. Its expression in both cancer cells and immune cells in the tumor microenvironment has been studied as a prognostic and predictive marker in a wide range of human malignancies [18,33]. Interestingly, in our series, PD-L1 expression was significantly higher in TSR high/stroma poor both in pre-operative biopsies and surgical specimens. This association between TSR and PD-L1 expression seems to be consistent with what has already been described in other tumor types, including colorectal cancer and spinal chordoma [34,35]. This association might be explained as follows: PD-1 secretion by mesenchymal stromal cells (which are highly represented in TRS low/stroma rich cases) may suppress T cell proliferation, leading to a reduction in their concentration, which also results in a downregulation of PD-L1 expression on the T cell surface [36]. According to current evidence, including PD-L1 expression on tumor infiltrating immune cells and not only on tumor cells increases the predictive value. In fact, CPS offers a more effective tumor evaluation than the tumor proportion score, which measures PD-L1 expression on tumor cells alone [37]. From a clinical viewpoint, a higher CPS has been regarded as a relevant predictive marker for targeted therapy, being associated with an enhanced response to PD-L1 inhibitors [16,38]. Accordingly, TSR, besides being a prognostic factor related with recurrence risk, also seems to have a potential role as a predictor of response to multi-kinase inhibitors in colon cancer [39]. This may be consistent with the biological pathways involved in tumor-to-stroma relation, resulting in downstream activation of multiple kinases, stimulated by the interaction between the stroma-secreted ligand HGF (Hepatocyte Growth Factor) and ‘onco-receptor’ MET (Hepatocyte Growth Factor Receptor) [40].

Despite the preliminary nature of our findings, the good concordance between biopsies and matched surgical specimens in TSR assessment may pave the way for the employment of this marker both in a preoperative risk assessment and as a predictor of response to a possible adjuvant therapy. Moreover, if confirmed by further data on a larger scale, the possibility of obtaining a reliable characterization of TSR from biopsies might allow us to use the latter as a predictive biological marker also in non-surgical settings, such as in advanced non-resectable cases, in a way similar to the current analysis of PD-L1 on biopsies in advanced head and neck cancers potentially suitable for checkpoint inhibitors [16,38]. Future prospective studies are mandatory before concluding that TSR and CPS—even evaluated in pre-treatment biopsies—could identify patients at higher risk of disease recurrence who might benefit from an intensification of the oncological treatment (neoadjuvant chemotherapy or postoperative radiotherapy or chemo-radiotherapy) or at least a closer clinical and radiological follow up.

This study has been the first that analyzed the integrated role of TSR and PD-L1 in a preoperative bioptic and final histopathological setting of LSCCs. The main strengths of this investigation lie in the homogeneity of the series of patients considered as: (I) a single specific histotype (SCC) located in a single head and neck structure (the larynx) was considered; (II) all patients underwent primary laryngeal surgery, performed consecutively by the same team in the same institution; (III) both preoperative and matched surgical samples of LSCC were assessed; (IV) various not conventional pathological variables were evaluated (TSR, stroma type, large cell nests and tumor budding, TILs); (V) the PD-L1 antibody used (22C3 IHC PharmDX) was a commercial clone that had been validated by a panel of experts for therapeutic purposes, as had the scoring system adopted (CPS); (VI) oncological follow-up clinical–radiological criteria were standardized. The main weaknesses of the study concern the retrospective nature of the investigation, and the limited number of cases considered, also due to the low number of cases with available preoperative biopsy.

## 4. Materials and Methods

### 4.1. Patients

The present study is a retrospective clinical investigation. No experimental diagnostic or therapeutic procedures have been applied; the procedures carried out corresponded to our normal expected clinical practice for LSCC. The study was conducted in accordance with the principles of the Helsinki Declaration. All patients signed a detailed informed consent form regarding the processing and publication of their data. They consented to “the use of their clinical data for scientific research purposes in the medical, biomedical and epidemiological fields, also in order to be recalled in the future for follow-up needs”. Data were examined in agreement with the Italian privacy and sensitive data laws, and the internal regulations of the University Hospital of Padova.

The study involved 43 cases of early and locally-advanced LSCC treated with primary surgery between 1999 and 2013. As in the recommendations adopted for LSCC at our institution, all patients (39 (90.7%) males, 4 (9.3%) females; mean age 65.0 ± 8.2 years) underwent microlaryngoscopy with laryngeal biopsy, upper aerodigestive tract endoscopy, neck ultrasonography (with or without fine needle aspiration cytology), head and neck contrast-enhanced computerized tomography (ceCT), and/or magnetic resonance imaging, chest X-ray and liver ultrasonography.

All patients underwent laryngeal surgery at our institution, including unilateral or bilateral cervical lymph node dissection in 41 cases (95.3%). Pathological findings warranted postoperative adjuvant RT (with or without concomitant chemotherapy) in 21 cases (48.8%) in accordance with current guidelines. No distant metastases (M) were detected at diagnosis. The clinical follow-up after treatment (adjustable to patients’ individual characteristics) was scheduled as follows: once a month in the 1st year; every 2 months in the 2nd year; every 3 months in the 3rd year; every 4 months in the 4th year; every 6 months in the 5th year; and every 12 months thereafter. ceCT of the neck, total body positron emission tomography, chest CT, and neck and liver ultrasonography were repeated if clinically indicated. The mean follow-up was 76.2 ± 46.5 months (median 80 months).

### 4.2. Histopathological Investigations

For each case, all H&E-stained slides from preoperative biopsies and paired surgical specimens available from the repository of the Pathology Department were revised by two head and neck pathologists (LA, MS). For each specific histopathological variable considered, the most representative slide of surgical specimens was selected. With regard to biopsy specimens, cases with insufficient tissue (less than 10 fields at 10×) or artifacts were excluded from the analysis. Using a 4× lens, the areas with the highest amount of stroma and including the invasive front of the tumor were selected. Subsequently, using a 10× lens, only the fields where both stroma and tumor were present and tumor cells were visualized on all sides of the field were assessed. TSR was defined as stroma poor/TSR high (with a proportion of stroma of <50%) or stroma rich/TSR low (with a proportion of stroma of ≥50%), as reported by van Pelt et al. [7]. As previously described [13], areas with necrosis were avoided, as well as major vascular structures and muscle tissue, whereas nerves, smaller vascular structures and lymphocytic infiltration were not excluded from the stromal compartment. Stroma, irrespective of its amount, was classified as fibroblastic when characterized by loose connective tissue or as fibrotic when a dense, homogeneous, collagenous stroma was present.

Tumor budding was defined as single cells or clusters of up to four cells at the invasive margin of the cancer; it was divided into peri-tumoral budding (PTB, tumor buds at the invasive front of the tumor), evaluated on surgical specimens’ slides, and intra-tumoral budding (ITB, tumor buds in the tumor core), evaluated on preoperative biopsies slides. To evaluate budding activity, 10 fields of the invasive front were scanned at medium magnification to identify the ‘hotspot’. The buds count in each hotspot was then divided by the most appropriate normalization factor (1.563), according to the eyepiece field number of the microscope used (25 mm), to ensure TB count per 0.785 mm^2^, as recommended by the ITBCC [41]. For risk-stratification purposes, a two-tier system to grade tumor buds count was applied, using a cut-off of five to discriminate between low risk (LR; less than 5 buds) and high risk (HR; 5 or more buds) cases, as frequently used in head and neck carcinoma [42]. The presence/absence of large neoplastic cell nests (defined as nests containing >15 neoplastic cells) was also recorded.

Growth patterns were defined as follows: expansive included tumors with well-circumscribed margins without normal tissue within the tumor and tumors with only few large neoplastic nests at the invasive front; infiltrative involved tumors with ill-circumscribed margins, interrupted by small nests and trabeculae of neoplastic tissue extending into normal tissue.

Tumor infiltrating lymphocytes (TILs) were scored as percentage of total peritumoral/intratumoral stroma occupied by mononuclear inflammatory cells (lymphocytes and plasma cells). These last two histological characteristics were evaluated only on slides from surgical specimens.

### 4.3. Immunohistochemistry

Immunohistochemistry (IHC) was performed using the Bond Polymer Refine Detection kit (Leica Biosystems, Newcastle upon Tyne, UK) in the BOND-MAX system (Leica Biosystems) with the primary antibody for PD-L1 (22C3, IHC PharmDx, Dako, Carpinteria, CA, USA; 1:50 dilution), as previously reported [15,17]. In detail, PD-L1 expression was assessed using CPS, which requires at least 100 viable neoplastic cells to be evaluated. CPS is calculated counting the number of PD-L1-positive cells (tumor cells, lymphocytes, and macrophages), dividing the result by the number of viable tumor cells, and multiplying by 100 [17]. CPS was determined according to manufacturer’s interpretation manual [43]. Cases were considered PD-L1-positive if the CPS was 1 or greater.

### 4.4. Statistical Analysis

The statistical analyses were performed with SAS 9.4 for Windows (SAS Institute Inc., Cary, NC, USA). The data are presented as means and standard deviations, medians and interquartile ranges for quantitative variables, and as counts and percentages for the categorical variables.

The concordance in TSR scoring between biopsy and surgical specimens was evaluated with the Gwet’s AC1 statistic, which expresses the conditional probability that two randomly selected raters agree given that there is not agreement by chance. It adjusts the overall agreement probability for chance agreement. Gwet’s AC1 statistic has the same interpretation of kappa statistic in the following range: <0.20, poor agreement; 0.21 to 0.40, fair agreement; 0.41 to 0.60, moderate agreement; 0.61 to 0.80, good agreement; 0.81 to 1.00, very good agreement.

The association of TSR, evaluated on biopsy and surgical specimens, with histopathological features was evaluated with Mann–Whitney test in the case of quantitative features, with Fisher’s exact test for categorical ones.

The prognostic role of each clinical–pathological and histopathological feature towards recurrence free was analyzed by applying univariate Cox regression. The disease-free survival (DFS) was calculated as the time from treatment completion to LSCC recurrence, or to last follow-up evaluation for censored patients.

The proportionality assumption of the Cox’s models was checked with a Kolmogorov-type supremum test using 1000 resampling. The results have been expressed as p-value and hazard-ratio (HR) with 95% confidence interval (CI). It was not possible to perform a multivariate Cox regression, since the variables resulted statistically significant at the univariate analysis, were associated.

A *p*-value < 0.05 was considered indicative of statistical significance.

## 5. Conclusions

The identification of new prognostic parameters is crucial for a better prognostic stratification of advanced LSCCs, possibly leading to the development of tailored therapeutic strategies. The present exploratory investigation found that TSR evaluated in pretreatment laryngeal biopsies and in the entire excised tumor displayed a prognostic effect. TSR is an easy-to-apply and time-sparing parameter that can be introduced in routine laryngological diagnostic practice, if validated in larger prospective series. Further investigations on the significant association between bioptic and surgical specimen TSR and PD-L1 expression may also lead to new insights into LSCC biology with a potential impact on targeted treatments, also with regard to the effectiveness of immunotherapeutic protocols based on programmed death-1 (PD-1)/PD-L1 checkpoint inhibitors.

## Figures and Tables

**Table 1:** Concordance between pathological variables measured on biopsies and on surgical specimens.

	Variables on Surgical Specimens			
	TSR	TSR High/Stroma Poor (N = 29)	TSR Low/Stroma Rich (N = 14)	AC1 Statistic (95% CI)
**TSR on biopsies**	TSR high/Stroma poor	27 (93.1%)	3 (21.4%)	0.7957 (0.6187–0.9727)
	TSR low/Stroma rich	2 (6.9%)	11 (78.6%)	
	**Stroma type**	**Fibroblastic** **(N = 5)**	**Fibrotic** **(N = 38)**	**AC1 statistic** **(95% CI)**
**Stroma type on biopsies**	Fibroblastic	3 (60.0%)	2 (5.3%)	0.8829 (0.7641–1.0000)
	Fibrotic	2 (40.0%)	36 (94.7%)	
	**Large cell nests**	**Absent** **(N = 8)**	**Present** **(N = 35)**	**AC1 statistic** **(95% CI)**
**Large cell nests on biopsies**	Absent	4 (50.0%)	5 (14.3%)	0.6935 (0.4849–0.9020)
	Present	4 (50.0%)	30 (85.7%)	
	**Tumor budding**	**Low risk** **(N = 37)**	**High risk** **(N = 6)**	**AC1 statistic** **(95% CI)**
**Tumor budding on biopsies**	Low risk	35 (94.6%)	4 (66.7%)	0.8244 (0.6768–0.9719)
	High risk	2 (5.4%)	2 (33.3%)	

TSR: tumor-to-stroma ratio; 95% CI: 95% confidence interval. Gwet’s AC1 statistic has the following interpretation: <0.20, poor agreement; 0.21 to 0.40, fair agreement; 0.41 to 0.60, moderate agreement; 0.61 to 0.80, good agreement; 0.81 to 1.00, very good agreement.

## Data Availability

The datasets generated and analyzed during the current study are available on reasonable request.

## References

[1] Hoffman H.T., Porter K., Karnell L.H., Cooper J.S., Weber R.S., Langer C.J., Ang K.-K., Gay G., Stewart A., Robinson R.A. (2006). Laryngeal cancer in the United States: Changes in demographics, patterns of care, and survival. Laryngoscope.

[2] Lucioni M., Marioni G., Bertolin A., Giacomelli L., Rizzotto G. (2011). Glottic laser surgery: Outcomes according to 2007 ELS classification. Eur. Arch. Oto-Rhino-Laryngol..

[3] Franz L., Tealdo G., Contro G., Bandolin L., Carraro V., Giacomelli L., Alessandrini L., Blandamura S., Marioni G. (2020). Biological tumor markers (maspin, CD105, nm23-H1) and disease relapse in laryngeal cancer: Cluster analysis. Head Neck.

[4] Marioni G., Ottaviano G., Lovato A., Franz L., Bandolin L., Contro G., Giacomelli L., Alessandrini L., Stramare R., De Filippis C. (2019). Expression of maspin tumor suppressor and mTOR in laryngeal carcinoma. Am. J. Otolaryngol..

[5] Lovato A., Franz L., Carraro V., Bandolin L., Contro G., Ottaviano G., de Filippis C., Blandamura S., Alessandrini L., Marioni G. (2020). Maspin expression and anti-apoptotic pathway regulation by bcl2 in laryngeal cancer. Ann. Diagn. Pathol..

[6] Pietras K., Östman A. (2010). Hallmarks of cancer: Interactions with the tumor stroma. Exp. Cell Res..

[7] Van Pelt G.W., Kjær-Frifeldt S., van Krieken J.H.J.M., Al Dieri R., Morreau H., Tollenaar R.A.E.M., Sørensen F.B., Mesker W.E. (2018). Scoring the tumor-stroma ratio in colon cancer: Procedure and recommendations. Virchows Arch..

[8] Wu J., Liang C., Chen M., Su W. (2016). Association between tumor-stroma ratio and prognosis in solid tumor patients: A systematic review and meta-analysis. Oncotarget.

[9] Almangush A., Alabi R.O., Troiano G., Coletta R.D., Salo T., Pirinen M., Mäkitie A.A., Leivo I. (2021). Clinical significance of tumor-stroma ratio in head and neck cancer: A systematic review and meta-analysis. BMC Cancer.

[10] Karpathiou G., Vieville M., Gavid M., Camy F., Dumollard J.M., Magné N., Froudarakis M., Prades J.M., Peoc’H M. (2019). Prognostic significance of tumor budding, tumor-stroma ratio, cell nests size, and stroma type in laryngeal and pharyngeal squamous cell carcinomas. Head Neck.

[11] Karpathiou G., Gavid M., Prevot-Bitot N., Dhomps A., Dumollard J.M., Vieville M., Lelonge Y., Prades J.M., Froudarakis M., Peoc’H M. (2020). Correlation between semiquantitative metabolic parameters after PET/CT and histologic prognostic factors in laryngeal and pharyngeal carcinoma. Head Neck Pathol..

[12] Zhang H., Sheng X., Zhang S., Gu X. (2020). The prognostic value of tumor budding in laryngeal squamous cell carcinoma. Transl. Cancer Res..

[13] Alessandrini L., Ferrari M., Taboni S., Sbaraglia M., Franz L., Saccardo T., Del Forno B.M., Agugiaro F., Frigo A.C., Tos A.P.D. (2022). Tumor-stroma ratio, neoangiogenesis and prognosis in laryngeal carcinoma. A pilot study on preoperative biopsies and matched surgical specimens. Oral Oncol..

[14] Alessandrini L., Zanoletti E., Cazzador D., Sbaraglia M., Franz L., Tealdo G., Frigo A.C., Blandamura S., Nicolai P., Mazzoni A. (2021). Tumor budding to investigate local invasion, metastasis and prognosis in temporal bone squamous cell carcinoma. Pathol. Res. Pract..

[15] Franz L., Alessandrini L., Ottaviano G., di Carlo R., Fasanaro E., Ramacciotti G., Contro G., Marioni G. (2020). Postoperative radiotherapy for laryngeal cancer. The prognostic role of programmed death-ligand 1: An immune microenvironment-based cluster analysis. Pathol. Res. Pract..

[16] Cohen E.E.W., Bell R.B., Bifulco C.B., Burtness B., Gillison M.L., Harrington K.J., Le Q.-T., Lee N.Y., Leidner R., Lewis R.L. (2019). The Society for Immunotherapy of Cancer consensus statement on immunotherapy for the treatment of squamous cell carcinoma of the head and neck (HNSCC). J. Immunother. Cancer.

[17] Franz L., Alessandrini L., Calvanese L., Crosetta G., Frigo A.C., Marioni G. (2021). Angiogenesis, programmed death ligand 1 (PD-L1) and immune microenvironment association in laryngeal carcinoma. Pathology.

[18] Burtness B., Harrington K.J., Greil R., Soulières D., Tahara M., de Castro G., Psyrri A., Basté N., Neupane P., Bratland A. (2019). Pembrolizumab alone or with chemotherapy versus cetuximab with chemotherapy for recurrent or metastatic squamous cell carcinoma of the head and neck (KEYNOTE-048): A randomised, open-label, phase 3 study. Lancet.

[19] Alessandrini L., Franz L., Ottaviano G., Ghi M.G., Lanza C., Blandamura S., Marioni G. (2020). Prognostic role of programmed death ligand 1 (PD-L1) and the immune microenvironment in laryngeal carcinoma. Oral Oncol..

[20] Altman D.G. (1991). Practical Statistics for Medical Research.

[21] Amin M.B. (2017). AJCC Staging Manual.

[22] Van Pelt G., Krol J., Lips I., Peters F., van Klaveren D., Boonstra J., de Steur W., Tollenaar R., Sarasqueta A.F., Mesker W. (2019). The value of tumor-stroma ratio as predictor of pathologic response after neoadjuvant chemoradiotherapy in esophageal cancer. Clin. Transl. Radiat. Oncol..

[23] Smit M.A., van Pelt G.W., Terpstra V., Putter H., Tollenaar R.A.E.M., Mesker W.E., van Krieken J.H.J.M. (2021). Tumour-stroma ratio outperforms tumour budding as biomarker in colon cancer: A cohort study. Int. J. Color. Dis..

[24] van Wyk H.C., Park J.H., Edwards J., Horgan P.G., McMillan D.C., Going J.J. (2016). The relationship between tumour budding, the tumour microenvironment and survival in patients with primary operable colorectal cancer. Br. J. Cancer.

[25] Franz L., Nicolè L., Frigo A., Ottaviano G., Gaudioso P., Saccardo T., Visconti F., Cappellesso R., Blandamura S., Fassina A. (2021). Epithelial-to-mesenchymal transition and neoangiogenesis in laryngeal squamous cell carcinoma. Cancers.

[26] Christofori G. (2006). New signals from the invasive front. Nature.

[27] Kim R., Emi M., Tanabe K. (2007). Cancer immunoediting from immune surveillance to immune escape. Immunology.

[28] A Gujam F.J., Edwards J., A Mohammed Z.M., Going J., McMillan D. (2014). The relationship between the tumour stroma percentage, clinicopathological characteristics and outcome in patients with operable ductal breast cancer. Br. J. Cancer.

[29] Zadka Ł., Chabowski M., Grybowski D., Piotrowska A., Dzięgiel P. (2021). Interplay of stromal tumor-infiltrating lymphocytes, normal colonic mucosa, cancer-associated fibroblasts, clinicopathological data and the immunoregulatory molecules of patients diagnosed with colorectal cancer. Cancer Immunol. Immunother..

[30] Jiang P., Gu S., Pan D., Fu J., Sahu A., Hu X., Li Z., Traugh N., Bu X., Li B. (2018). Signatures of T cell dysfunction and exclusion predict cancer immunotherapy response. Nat. Med..

[31] Ravensbergen C.J., Polack M., Roelands J., Crobach S., Putter H., Gelderblom H., Tollenaar R.A.E.M., Mesker W.E. (2021). Combined assessment of the tumor–stroma ratio and tumor immune cell infiltrate for immune checkpoint inhibitor therapy response prediction in colon cancer. Cells.

[32] Ravensbergen C.J., Kuruc M., Polack M., Crobach S., Putter H., Gelderblom H., Roy D., Tollenaar R.A.E.M., Mesker W.E. (2021). The stroma liquid biopsy panel contains a stromal-epithelial gene signature ratio that is associated with the histologic tumor-stroma ratio and predicts survival in colon cancer. Cancers.

[33] Zhuang Y., Liu C., Liu J., Li G. (2020). Resistance mechanism of PD-1/PD-L1 blockade in the cancer-immunity cycle. Onco Targets Ther..

[34] Mohamed A.S.E.D., El-Rebey H.S., AboElnasr L.S.a., Abdou A.G. (2021). The role and relationship between programmed death ligand 1 and cytotoxic T lymphocyte-associated antigen-4 immunohistochemical expression in colorectal carcinoma patients; an impact on outcome. Ecancermedicalscience.

[35] Zou M.-X., Zheng B.-W., Liu F.-S., Wang X.-B., Hu J.-R., Huang W., Dai Z.-H., Zhang Q.-S., Liu F.-B., Zhong H. (2019). The relationship between tumor-stroma ratio, the immune microenvironment, and survival in patients with spinal chordoma. Neurosurgery.

[36] Davies L.C., Heldring N., Kadri N., Le Blanc K. (2016). Mesenchymal stromal cell secretion of Programmed Death-1 ligands regulates T cell mediated immunosuppression. Stem Cells.

[37] Fasano M., Della Corte C.M., Di Liello R., Viscardi G., Sparano F., Iacovino M.L., Paragliola F., Piccolo A., Napolitano S., Martini G. (2022). Immunotherapy for head and neck cancer: Present and future. Crit. Rev. Oncol..

[38] Cramer J.D., Burtness B., Ferris R.L. (2019). Immunotherapy for head and neck cancer: Recent advances and future directions. Oral Oncol..

[39] Takigawa H., Kitadai Y., Shinagawa K., Yuge R., Higashi Y., Tanaka S., Yasui W., Chayama K. (2016). Multikinase inhibitor regorafenib inhibits the growth and metastasis of colon cancer with abundant stroma. Cancer Sci..

[40] Modica C., Tortarolo D., Comoglio P.M., Basilico C., Vigna E. (2018). MET/HGF co-targeting in pancreatic cancer: A tool to provide insight into the tumor/stroma crosstalk. Int. J. Mol. Sci..

[41] Lugli A., Kirsch R., Ajioka Y., Bosman F., Cathomas G., Dawson H., El Zimaity H., Fléjou J.-F., Hansen T.P., Hartmann A. (2017). Recommendations for reporting tumor budding in colorectal cancer based on the International Tumor Budding Consensus Conference (ITBCC) 2016. Mod. Pathol..

[42] Almangush A., Salo T., Hagström J., Leivo I. (2014). Tumour budding in head and neck squamous cell carcinoma—A systematic review. Histopathology.

[43] PD-L1 IHC 22C3 PharmDx Interpretation Manual—Head and Neck Squamous Cell Carcinoma (HNSCC). https://www.agilent.com/cs/library/usermanuals/public/29314_22c3_phamDx_hnscc_interpretation_manual_us.pdf.

